# A Robust Sparse Representation Model for Hyperspectral Image Classification †

**DOI:** 10.3390/s17092087

**Published:** 2017-09-12

**Authors:** Shaoguang Huang, Hongyan Zhang, Aleksandra Pižurica

**Affiliations:** 1 Department of Telecommunications and Information Processing, Ghent University, Sint Pietersnieuwstraat 41, 9000 Gent, Belgium; aleksandra.pizurica@ugent.be; 2 The State Key Laboratory of Information Engineering in Surveying, Mapping, and Remote Sensing, Wuhan University, Luoyu Road 129, Wuhan 430079, China; zhanghongyan@whu.edu.cn

**Keywords:** robust classification, hyperspectral image, super-pixel segmentation, sparse representation

## Abstract

Sparse representation has been extensively investigated for hyperspectral image (HSI) classification and led to substantial improvements in the performance over the traditional methods, such as support vector machine (SVM). However, the existing sparsity-based classification methods typically assume Gaussian noise, neglecting the fact that HSIs are often corrupted by different types of noise in practice. In this paper, we develop a robust classification model that admits realistic mixed noise, which includes Gaussian noise and sparse noise. We combine a model for mixed noise with a prior on the representation coefficients of input data within a unified framework, which produces three kinds of robust classification methods based on sparse representation classification (SRC), joint SRC and joint SRC on a super-pixels level. Experimental results on simulated and real data demonstrate the effectiveness of the proposed method and clear benefits from the introduced mixed-noise model.

## 1. Introduction

Unlike classical multispectral images, hyperspectral images (HSIs) provide richer spectral information about the image objects in hundreds of narrow bands. A HSI is captured as a three-dimensional data cube comprising two-dimensional spatial information and one-dimensional spectral information. The spectral signature of a pixel is a vector whose entries correspond to spectral responses in different bands. Different materials have diverse spectral signatures, thus hyperspectral imaging allows differentiation between materials that are often visually indistinguishable. Numerous application areas include agriculture [1,2], defense and security [3] and environmental monitoring [4,5].

Classification of HSIs currently enjoys huge interest in the remote sensing community. The objective of supervised hyperspectral classification is to group pixels into different classes with the classifiers trained by the given training samples. A large number of HSI classification methods have been proposed, based on artificial neural networks [6], multinomial logistic regression [7,8], spectral-spatial preprocessing with multihypothesis prediction [9], information fusion [10] and support vector machines (SVM) [11], just to name a few. With the target of exploiting spatial information in the classification task, spatial-spectral classification approaches have been developed, including SVM with composite kernels [12], methods based on mathematical morphology [13,14,15,16,17] and image segmentation [18].

In recent years, sparse representation classification (SRC) [19] emerged as another effective classification approach, which became widely adopted for HSI [20,21,22,23,24,25,26,27,28,29,30,31,32,33,34]. SRC assumes that each test sample can be sparsely represented as a linear combination of atoms from a dictionary, which is constructed or learned from training samples [19]. Chen et al. [20] introduced the joint sparse representation classification (JSRC) in HSI classification by incorporating spatial information. The model was based on the observation that the pixels in a patch share similar spectral characteristics and can be represented by a common set of atoms but with different sparse coefficients. Zhang et al. [21] proposed a nonlocal weighted joint sparse representation (NLW-JSRC) to further improve the classification accuracy. They enforced a weight matrix on the pixels of a patch in order to discard the invalid pixels whose class was different from that of the central pixel. The works in [22,24] extended the JSRC to the kernel versions to address the linearly non-separable problem. In [27], a multi-layer spatial-spectral sparse representation framework was proposed for HSI classification in order to stabilize the sparse codes of the traditional single-layer sparse representation. Related classification methods effectively exploiting spatial information with adaptive neighborhood were reported in [25,26,31] and produced good results. Recent studies in [29,30,31,32,33] indicated that learning a compact and discriminative dictionary from the training samples can reduce the computational burden significantly.

However, all of these sparsity-based methods for HSI classification only take into account Gaussian noise. In real applications, HSIs are inevitably corrupted by different kinds of noise, including Gaussian noise and sparse noise. Here, sparse noise is defined as the noise of arbitrary magnitude that only affects certain bands or pixels, which can be impulse noise, dead lines and strips. It may arise due to the defective pixels and poor imaging conditions such as water vapor and atmospheric effect [35]. With the consideration of sparse noise in the tasks of HSIs denoising [36,37], unmixing [35,38] and robust learning [39], significant improvements have been achieved over the state-of-the-art methods, which indicates the importance of taking the sparse noise into account in those tasks. For the classification task, the sparse noise can hinder the performance undoubtedly. We are not aware of any sparsity-based classification method that takes it explicitly into account. This motivates us to develop a robust classification model that accounts for realistic degradations in the HSIs.

The key idea of our model is to incorporate the presence of sparse noise in HSIs into the classification problem, by combining the appropriate statistical models for the sparse noise and the representation coefficients of test pixel(s) within a unified framework. In particular, we make use of the fact that test pixels can be represented with relatively few atoms from a well constructed dictionary, meaning that the representation coefficients are sparse or jointly sparse within small neighborhoods. This is the main assumption of SRC and JSRC models. In addition, we introduce a statistical model for the sparse noise as an instance of a multivariate Laplacian distribution, which allows us to derive an optimization problem that extends elegantly the previous ones with an additional $\ell_{1}$ norm on the sparse noise term. Following this idea, we extend and generalize the existing SRC [19] and JSRC [20] methods to the robust versions, i.e., robust SRC (R-SRC) and robust JSRC (R-JSRC), respectively. We also derive an optimization algorithm for the corresponding objective function, based on the alternating minimization strategy.

Moreover, in order to further exploit the available spatial information, we extend the R-JSRC model to a classification model on a super-pixel level. In the JSRC model, spatial information is defined by the collection of neighbouring pixels in a square window of fixed size, while super-pixel segmentation can adaptively divide the HSIs into a number of non-overlapping homogenous regions depending on the spatial content, which makes the joint sparse representation more effective and precise. We name this extended method robust super-pixel level joint sparse representation classification (R-SJSRC). The results on simulated and real data demonstrate improved performance in comparison to recent related methods and a clear benefit resulting from the introduced robust model. Parts of this work have been accepted for presentation at a conference [40]. In comparison to the conference version, here we give more elaborate presentation and analysis of the method. Moreover, extra experiments with both simulated and real HSI data are conducted to investigate the effect of sparse noise and parameters on performance.

The main contributions of the paper can be summarized as follows:

(1) A robust sparsity-based classification model for HSIs is proposed when the data is corrupted by Gaussian noise and sparse noise, by incorporating the appropriate priors for noise-free data and degradations into an optimization framework.
(2) An efficient algorithm is developed to solve the optimization problem by using an alternating minimization strategy.
(3) The robust model is extended to efficiently incorporate spatial information. By jointly processing super-pixels, we strongly improve the performance both in terms of the classification accuracy and processing speed.

The rest of this paper is organized as follows. Section 2 reviews briefly the classical sparsity-based models in HSI classification. Section 3 extends the existing sparsity-based models to the robust versions and designs an effective algorithm to solve corresponding optimization problems. Section 4 presents experimental results with simulated and real data and Section 5 concludes the paper.

## 2. Sparsity-Based Models in HSI Classification

### 2.1. Sparse Representation Classification

Let $x\in R^{B}$ be a test pixel and $D=\left[D_{1},D_{2},...,D_{C}\right]\in R^{B\times d}$ a structured dictionary constructed from training samples, where *B* is the number of bands in the HSI; *d* is the number of training samples; *C* is the number of classes, and $D_{i}\in R^{B\times d_{i}}$ (*i* = 1, 2, ..., *C*) is the sub-dictionary in which each column is a training sample of *i*-th class, and $d_{i}$ is the number of training samples from class *i*, such that $\sum_{i=1}^{C}d_{i}=d$. The goal of sparse representation is to represent each test pixel as

(1) $$\begin{array}{c} x=D\alpha +n, \end{array}$$

where $n\in R^{B}$ is Gaussian noise and $\alpha \in R^{d}$ are sparse coefficients, satisfying

(2) $$\begin{array}{c} \hat{\alpha }=\underset{\alpha }{\arg\min}{∥x-D\alpha ∥}_{2}^{2}\;\;\;\;\;s.t.\;\;\;\;\;{∥\alpha ∥}_{0}\leq K. \end{array}$$

${∥\alpha ∥}_{0}$ denotes the number of non-zero elements in α and *K* is the sparsity level, i.e., the largest number of atoms in dictionary D needed to represent any input sample x. Problem in Equation (2) is typically solved with a greedy algorithm, such as Orthogonal Matching Pursuit (OMP) [41].

The class of the test sample is identified by calculating the class-specific residuals $r_{i}$ [19]:

(3) $$\begin{array}{ll} class(x) & =\underset{i=1,2,...,C}{\arg\min}r_{i}\left(x\right)\\ & =\underset{i=1,2,...,C}{\arg\min}{∥x-D_{i}\alpha_{i}∥}_{2}, \end{array}$$

where $\alpha_{i}$ are the sparse coefficients associated with class *i*.

### 2.2. Joint Sparse Representation Classification

An effective method to exploit the spatial information of the HSI is using joint sparse representation of neighbouring pixels. The assumption is that the pixels in a small patch are likely to belong to the same class and thus share the same sparsity pattern, meaning that they can be represented by the same set of atoms but with different sets of coefficients [20]. In the JSRC model, the spatial neighbourhood for the central pixel is a square window and all the neighbouring pixels are gathered into the input matrix $X=\left[x_{1},x_{2},...,x_{T}\right]\in R^{B\times T}$, where $x_{i}$ is the spectral signature of the *i*-th pixel in a patch of size $\sqrt{T}\times \sqrt{T}$. Denoting by $\alpha_{i}$ the sparse coefficients of $x_{i}$ in dictionary D leads to

(4) $$\begin{array}{cc} X & =[x_{1},x_{2},...,x_{T}]\\ & =[D\alpha_{1}+n_{1},D\alpha_{2}+n_{2},...,D\alpha_{T}+n_{T}]\\ & =DA+N, \end{array}$$

where $A=\left[\alpha_{1},\alpha_{2},...,\alpha_{T}\right]\in R^{d\times T}$ is the coefficient matrix.

Since all $x_{i}$ in a small patch are likely to belong to the same class and thus share the same set of atoms, $\alpha_{i}$ have non-zero entries at the same positions. Therefore, A is row-sparse, and can be obtained by solving the following problem with Simultaneous Orthogonal Matching Pursuit (SOMP) algorithm [42]:

(5) $$\begin{array}{c} \hat{A}=\underset{A}{\arg\min}{∥X-DA∥}_{F}^{2}\\ {s.t.\;∥A∥}_{row,0}\leq K_{0},\;\;\;\;\;\;\; \end{array}$$

where ${∥X∥}_{F}$ denotes the Frobenius norm of X, ${∥A∥}_{row,0}$ denotes the number of non-zero rows of A and $K_{0}$ is the row-sparsity level. In a similar way to SRC, the central test pixel of the patch is labeled by minimizing the class-specific residual:

(6) $$\begin{array}{c} class\left(x_{central}\right)=\underset{i=1,2,...,C}{\arg\min}{∥X-D_{i}A_{i}∥}_{F}, \end{array}$$

where $A_{i}$ is the portion of the sparse matrix A associated with class *i*.

## 3. Proposed Method

### 3.1. Robust SRC Model

**Figure 1:**
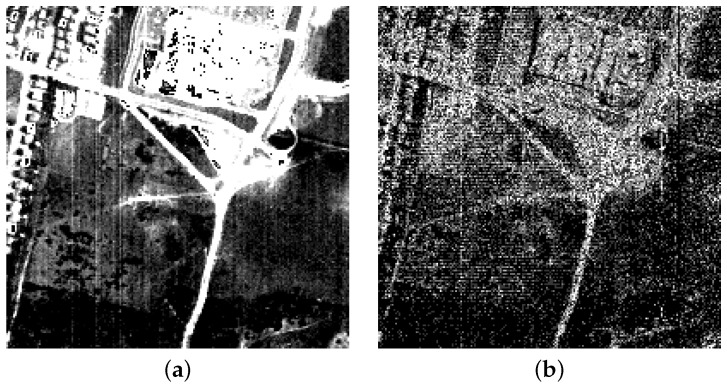
An illustration of noise in a real HSI. Two bands from HYDICE Urban data set are shown. (**a**) sparse noise (vertical lines) in band 2 (contrast enhanced); (**b**) mixed noise in band 206.

Here, we develop a more general classification method, which takes into account not only the Gaussian noise (as described above) but also sparse noise, which affects real HSIs. The motivation is as follows. In practice, HSIs are often contaminated by horizontal and vertical strips, impulse noise and dead lines. This type of degradation is called sparse noise as it affects only relatively few pixels. Sparse noise typically arises in situations with poor imaging conditions due to sensor artifacts. In the real HSIs, different bands can be corrupted by different kinds of noise [35,38]. In some bands, sparse noise is a dominant degradation, while others may be corrupted by mixed noise. An example of the noise in real HSI (Hyperspectral Digital Image Collection Experiment (HYDICE) Urban data set [35]) can be found in Figure 1, where Figure 1a shows a band affected with stripe noise, and Figure 1b shows a band affected by a mixture of sparse noise and Gaussian noise. We model the observed pixel in HSI as:

(7) $$\begin{array}{c} x=y+s+n, \end{array}$$

where $y\in R^{B}$ is an error-free sample, $s\in R^{B}$ sparse noise and $n\in R^{B}$ Gaussian noise.

As the error-free samples are not available in practice, we have to express y in terms of the observed samples. To this end, we will employ in our derivation a hypothetic, ideal dictionary $D^{y}$. Let $D^{y}\in R^{B\times d}=\left[y_{1},y_{2},...,y_{d}\right]$ denote an ideal, error-free dictionary and $y_{j}\in R^{B}$ the *j*-th error-free training sample. The main assumption of SRC is that any y can be represented by a few atoms in $D^{y}$ as follows:

(8) $$\begin{array}{c} y=D^{y}\alpha +\varepsilon , \end{array}$$

where α is a sparse vector and ε is arbitrarily small.

The model (7) holds for any observed sample: $x_{i}=y_{i}+s_{i}+n_{i}$. Equivalently, we can write

(9) $$\begin{array}{c} D=D^{y}+D^{s}+D^{n}, \end{array}$$

where $D=[x_{1},x_{2},...,x_{d}]$, $D^{s}=\left[s_{1},s_{2},...,s_{d}\right]$ and $D^{n}=\left[n_{1},n_{2},...,n_{d}\right]$ are collections, or dictionaries, composed of the observed data $x_{i}$, sparse noise components $s_{i}$ and Gaussian noise components $n_{i}$, respectively.

Substituting the Equations (7) and (9) into (8), we derive the representation of x as follows:

(10) $$\begin{array}{cc} x & =(D-D^{s}-D^{n})\alpha +\varepsilon +s+n\\ & =D\alpha +s^{\prime }+n^{\prime }, \end{array}$$

where $s^{\prime }=s-D^{s}\alpha$ and $n^{\prime }=n-D^{n}\alpha +\varepsilon$.

A linear combination of two (or more) sparse vectors is not necessarily sparse. However, the sparse noise in HSIs is typically detected only in certain and relatively few bands, which means the non-zero elements of s and $s_{i}$ are located at the relatively few positions. Therefore $s^{\prime }$, being a linear combination of s and elements of ${\left\{s_{i}\right\}}_{i=1}^{d}$, is sparse as well. The expression in Equation (10) tells us that the observed pixel contaminated by sparse noise and Gaussian noise can be represented by relatively few atoms from the noisy dictionary with the addition of a sparse term $s^{\prime }$ and an error term $n^{\prime }$. Note that here $s^{\prime }$ in Equation (10) is not exactly the sparse noise of x but a mixture of the sparse noise in x and D, which is the reason why this model can not be directly used in the denoising task.

Now, we are ready to define an optimization problem that generalizes the one in Equation (2) as a result of our mixed-noise model. Observe first that the problem in Equation (2) can equivalently be written as

(11) $$\begin{array}{c} \underset{\alpha }{\arg\max}p\left(x;\alpha \right)\;\;\;\;\;\;s.t.\;\;\;\;\;{∥\alpha ∥}_{0}<K, \end{array}$$

where p(x;α) is the probability distribution of x with parameter α, which is according to the model in Equation (1): $p\left(x;\alpha \right)=N\left(D\alpha ;\sigma_{n}I\right)\propto exp(-\frac{1}{2\sigma_{n}^{2}}{∥x-D\alpha ∥}_{2}^{2})$. We formulate a similar problem taking into account the sparse noise $s^{\prime }$:

(12) $$\begin{array}{c} \underset{\alpha ,s^{\prime }}{\arg\max}p\left(x,s^{\prime };\alpha \right)\;\;\;\;\;\;s.t.\;\;\;\;\;{∥\alpha ∥}_{0}<K. \end{array}$$

Making use of the fact that $p\left(x,s^{\prime }\right)=p\left(x|s^{\prime }\right)p\left(s^{\prime }\right)$ and that the parameter α appears only in the first term, we can rewrite the objective function in Equation (12) as

(13) $$\begin{array}{c} \underset{\alpha ,s^{\prime }}{\arg\max}p\left(x|s^{\prime };\alpha \right)p\left(s^{\prime }\right)\;\;\;\;\;\;s.t.\;\;\;\;\;{∥\alpha ∥}_{0}<K. \end{array}$$

From our model (10), it follows that $p\left(x|s^{\prime };\alpha \right)=N\left(D\alpha +s^{\prime };\sigma_{n^{\prime }}I\right)\propto exp(-\frac{1}{2\sigma_{n^{\prime }}^{2}}∥x-D\alpha -s^{\prime }{∥}_{2}^{2})$. By imposing a Laplacian prior on $s^{\prime }$ of the form: $p\left(s^{\prime }\right)\propto exp(-\frac{1}{2\tau }∥s^{\prime }{∥}_{1})$ with τ>0 and $∥s^{\prime }{∥}_{1}=\sum_{i=1}^{B}\left|s_{i}^{\prime }\right|$, the left-hand term in Equation (13) can be written as

(14) $$\begin{array}{c} \underset{\alpha ,s^{\prime }}{\arg\min}\frac{1}{2\sigma_{n^{\prime }}}∥x-D\alpha -s^{\prime }{∥}_{2}^{2}+\frac{1}{2\tau }{∥s^{\prime }∥}_{1}. \end{array}$$

With this, we can rewrite the Equation (13) as

(15) $$\begin{array}{c} \underset{\alpha ,s^{\prime }}{\arg\min}∥x-D\alpha -s^{\prime }{∥}_{2}^{2}+\lambda ∥s^{\prime }{∥}_{1}\;\;\;\;\;\;\;s.t.\;\;\;\;\;{∥\alpha ∥}_{0}\leq K,\;\;\; \end{array}$$

where $\lambda =\sigma_{n^{\prime }}/\tau$ is a positive parameter that controls the tradeoff between data fidelity and the constraint on the sparse noise.

The resulting optimization problem in Equation (15) combines a prior knowledge about the representation coefficients α (meaning that α is sparse), a statistical model for the observation x in Equation (7) expressed as $x∼N(D\alpha +s^{\prime };\sigma_{n^{\prime }}I)$, and a prior model for the sparse noise $s^{\prime }\propto exp(-\frac{1}{2\tau }∥s^{\prime }{∥}_{1})$. We solve this problem by an alternating minimization algorithm described later (Section 3.4).

Once the sparse coefficients are obtained, we can calculate the class of x by

(16) $$\begin{array}{c} class\left(x\right)=\underset{i=1,2,...,C}{\arg\min}{∥x-D_{i}\alpha_{i}-s^{\prime }∥}_{2}, \end{array}$$

where $\alpha_{i}$ is a sparse vector associated with class *i*.

### 3.2. Robust JSRC Model

Similar to Equation (4), by gathering all the neighbouring pixels around a central test pixel into a matrix X, we can rewrite the Equation (10) in matrix form as follows:

(17) $$\begin{array}{c} X=DA+S+N, \end{array}$$

where $S\in R^{B\times T}$ and $N\in R^{B\times T}$ are the corresponding matrices representing sparse noise and Gaussian noise, respectively. With the assumption as in the JSRC model that the pixels in a small patch share the same set of training samples, the proposed optimization problem with respect to A and S can be formulated as:

(18) $$\begin{array}{c} \min f\left(A,S\right)=\min_{A,S}{∥X-DA-S∥}_{F}^{2}+\lambda {∥S∥}_{1}\;\;\\ {s.t.\;∥A∥}_{row,0}\leq K_{0},\;\;\;\;\;\;\;\;\;\;\;\;\; \end{array}$$

where ${∥S∥}_{1}$ is a norm defined as ${∥S∥}_{1}=\sum_{i,j}\left|S_{i,j}\right|$.

After finding the sparse coefficient matrix A and the sparse noise matrix S, we can label the class of the central pixel by

(19) $$\begin{array}{c} class\left(x_{central}\right)=\underset{i=1,2,...,C}{\arg\min}{∥X-D_{i}A_{i}-S∥}_{F}, \end{array}$$

where $A_{i}$ denotes the sparse matrix of A corresponding to class *i*.

### 3.3. Robust Super-Pixel Level JSRC

Imposing that pixels within a fixed-size rectangular neighbourhood share the same sparsity pattern, as in JSRC, has the following limitations. First, the size of the window is a free parameter, and determining its optimal value requires some tuning that varies from one image to the other. Secondly, when the central pixel is located on or near the boundaries between different classes, its neighbouring pixels belong to multiple classes, violating the assumption of the JSRC model and causing classification errors in these border regions. Finally, in practice, both the shape and the size of nearly homogeneous regions may vary a lot across a real scene, which suggests adaptive neighbourhoods instead of the fixed ones. The price to pay for such adaptive instead of fixed neighbourhoods is that a certain type of segmentation is needed. However, it turns out that such an approach with adaptive neighbourhoods may be advantageous not only in terms of accuracy, but also in terms of the net computation time, since each small region can be classified simultaneously as we show next.

We develop here a robust JSRC model, where the spatial information is captured at a super-pixel level, instead of using fixed-size rectangular neighbourhoods. Super-pixel segmentation techniques [43] adaptively divide the image into non-overlapping super-pixels being nearly homogeneous regions according to some criterion. In our problem, each super-pixel is a relatively small arbitrarily shaped and nearly homogeneous region, composed of pixels that belong to the same class. Let X now denote a matrix composed of pixels within the same super-pixel. With the same reasoning as in the previous section, we assume the model in Equation (17). Note that now the size of X is not fixed, but, otherwise, the formal description remains equivalent to the previous one, with the optimisation problem defined in Equation (18).

An important difference, both formally and practically, is that now we can assign X simultaneously to a given class instead of its central pixel alone in Equation (19). Now, we have

(20) $$\begin{array}{c} class\left(X\right)=\underset{i=1,2,...,C}{\arg\min}{∥X-D_{i}A_{i}-S∥}_{F}. \end{array}$$

Here, the class label of a super-pixel is simultaneously calculated, which means also that the sparse coding problem, calculation of class residuals and the minimization over these is calculated only once per non-overlapping super-pixel. On the contrary, in Section 3.2, all these operations are performed in each sliding window, centred around each image pixel. A typical hyperspectral image in remote sensing often has the size of thousands by thousands or more amounting to over million pixels, while we segment it into a couple of hundreds or thousands of super-pixels. This indicates a tremendous saving in computation. The concrete example are given in Section 4.2.

### 3.4. Optimization Algorithm

Here, we present an optimization algorithm to solve the proposed robust model by an alternating minimization strategy. A general derivation for the optimization in a matrix form is shown in Algorithm 1, where the input matrix X can represent a patch in R-JSRC or a super-pixel in R-SJSRC or reduce to a single vector in R-SRC. We employ alternating minimization similarly as in [28,31,36,44] to split a difficult problem into two easily solvable ones by fixing one variable in the other sub-problem, and alternating the process iteratively. In the (k+1)th iteration, we update A and S as follows:

(21) $$\begin{array}{c} A^{\left(k+1\right)}=\underset{{∥A∥}_{row,0}\leq K_{0}}{\arg\min}f\left(A,S^{\left(k\right)}\right), \end{array}$$

(22) $$\begin{array}{c} S^{\left(k+1\right)}=\underset{S}{\arg\min}f\left(A^{\left(k+1\right)},S\right). \end{array}$$

Problem in Equation (21) can be solved by the SOMP algorithm [42], and for problem in Equation (22), the optimization with respect to $S^{\left(k+1\right)}$ is formulated by

(23) $$\begin{array}{c} \min_{S}∥X-DA^{\left(k+1\right)}{-S∥}_{F}^{2}+{\lambda ||S||}_{1}, \end{array}$$

which is the well-known shrinkage problem. By introducing the following soft-thresholding operator:

(24) $$\begin{array}{c} {ℜ}_{\Delta }\left(x\right)=\left\{\begin{array}{cc} sgn(x)(|x|-\Delta ), & if\;|x|\geq \Delta ,\\ 0, & if\;|x|<\Delta , \end{array}\right. \end{array}$$

the solution of Equation (23) could be given by

(25) $$\begin{array}{c} S^{\left(k+1\right)}={ℜ}_{\lambda /2}\left(X-{\mathrm{DA}}^{\left(k+1\right)}\right). \end{array}$$

Note that, for the vector form of R-SRC in Algorithm 1, the sparse coefficients α in step 4 are obtained by OMP algorithm [41] and $s^{\prime }$ in step 5 is derived by ${ℜ}_{\lambda /2}\left(x-D\alpha \right)$. The class in step 8 is labeled by Equation (16).

**Algorithm 1** Generic pseudo-code of the proposed approach
1:**Input**: input matrix **X**, dictionary **D**, $K_{0}$ and λ  2:**Initialize**: $A^{\left(0\right)}=0$, $S^{\left(0\right)}=0$ and k=0   3:**While** stop criterion is not satisfied **do**4:   Obtain $A^{\left(k+1\right)}$ by solving the sub-problem in Equation (21)5:   Obtain $S^{\left(k+1\right)}$ by Equation (25)   6:**end**7:**Return**: $A=A^{\left(k+1\right)}$, $S=S^{\left(k+1\right)}$   8:**Output**: Class label is obtained by residuals (Equation (19) for R-JSRC and Equation (20) for R-SJSRC).


## 4. Experiments

We evaluate the performance of our methods on both simulated and real hyperspectral images, in comparison with SVM with radial basis function (RBF) kernel [45], SRC [19], JSRC [20] and NLW-JSRC [21]. As quantitative performance measures, we adopt the common indicators: overall accuracy (OA), average accuracy (AA) and Kappa coefficient (κ). All the reported results represent the average of ten runs. In each run, the training samples are randomly selected and the remaining labeled samples are used for testing.

### 4.1. Results for the Simulated HSI Experiment

**Figure 2:**
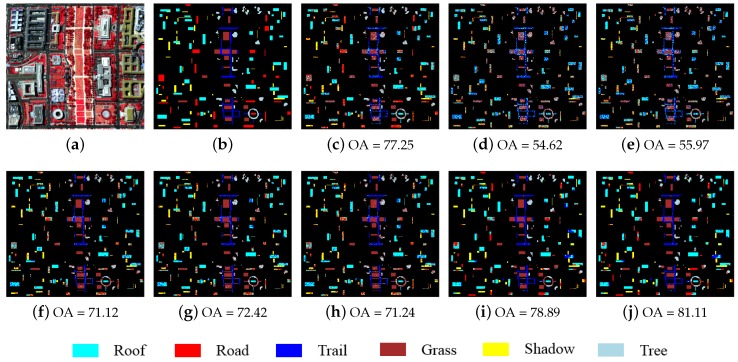
Washington DC image. (**a**) false color image; (**b**) ground truth; and classification results (OA in percentage) obtained by (**c**) SVM; (**d**) SRC; (**e**) R-SRC; (**f**) JSRC; (**g**) R-JSRC; (**h**) NLW-JSRC; (**i**) SJSRC; (**j**) R-SJSRC.

The Washington DC image shown in Figure 2a was collected by the HYDICE. Due to its high quality, this image is commonly used to simulate data degraded with different kinds of noise. The image is of size 280×307×210 with the spectrum ranging from 0.4 to 2.4 μm and has six classes in total. In this experiment, we reduce the number of bands to 191 by removing the opaque bands. Five percent of labeled samples were randomly selected as training samples and the remainder as test samples as shown in Table 1.

*Experiment 1 (Synthetic simulation)*: In this simulated experiment, four kinds of noise were added as follows:

1. Zero-mean Gaussian noise in all bands with SNR value for each band varying from 10 to 20 dB.
2. Impulse noise with 20% of corrupted pixels in bands 30–40.
3. Dead lines in bands 70–73 with width ranging from one line to three lines.
4. Strips in bands 101–104 with width ranging from one line to three lines.

The optimal parameters of the proposed robust methods were determined empirically as: λ=0.01, K=5 for the R-SRC model, $\lambda =0.02,K_{0}=30,T=25$ for the R-JSRC model and λ=0.02, $K_{0}=30,Ns=7000$ for the R-SJSRC model, where Ns denotes the number of super-pixels. For other classification methods in Table 1, all the parameters were tuned to give the best results, which are denoted in bold and suboptimal results are underlined. In order to be able to evaluate the contribution of each of the components of the proposed approach separately (both the robust nature and handling of spatial context), we also implemented the super-pixel level joint sparse representation classification (SJSRC) method with the same segmentation map as R-SJSRC. The results in Table 1 and Figure 2 show that the R-SJSRC model yielded a superior performance in terms of OA, AA and Kappa coefficient. The improvement due to the better spatial modelling can be clearly seen by comparing the performance of the super-pixel based SJSRC with the original JSRC. In terms of OA, this improvement was above 9.6%. Further improvement in the performance results from the improved noise model in R-SJSRC (the OA increases by other 1.5% compared to SJSRC). Similarly, the robust versions R-SRC and R-JSRC improve consistently over the corresponding SRC and JSRC methods, respectively.

**Figure 3:**
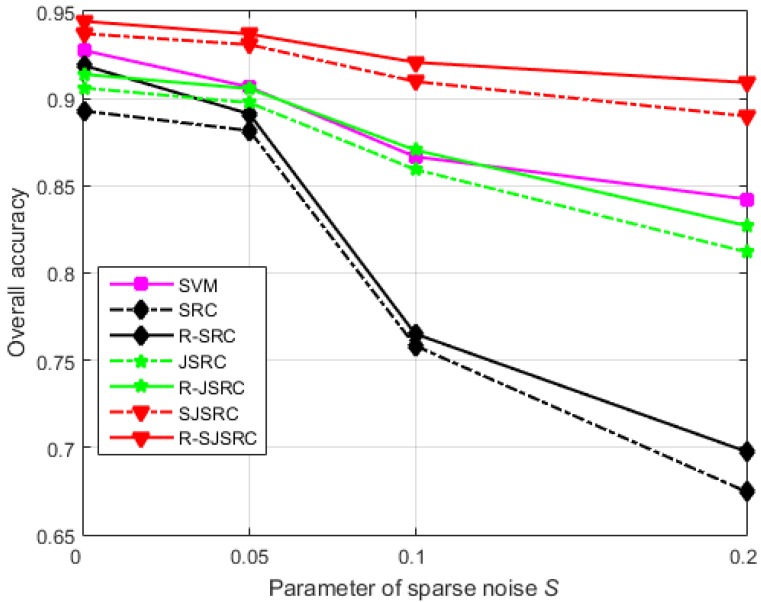
The influence of sparse noise on the classification performance of different classifiers. The overall accuracy is plotted against the parameter *S* reflecting the level of sparse noise.

*Experiment 2 (Effect of sparse noise)*: In this experiment, we analyse the robustness of our models to degradations dominated by sparse noise. We attempt to simulate a realistic situation where at least a small amount of Gaussian noise is always present and where sparse noise only affects certain bands or pixels of HSIs. Therefore, we first add a small amount of zero mean white Gaussian noise, such that the resulting SNR is 30 dB, and subsequently we introduce sparse noise. Let $S_{b}$ denote the fraction of bands affected by sparse noise and $S_{p}$ the fraction of affected pixels in each band. We perform experiments with $S_{b}=S_{p}=S\in \left\{0,0.05,0.1,0.2\right\}$. The results are reported in Figure 3. R-SJSRC is the most stable method among all the tested ones, while SRC degrades sharply with the increasing level of sparse noise. Clearly, the performance of R-SJSRC is less sensitive to sparse noise than that of other methods. Moreover, the robust methods R-SRC, R-JSRC and R-SJSRC yield consistent improvements over the original models as expected.

**Figure 4:**
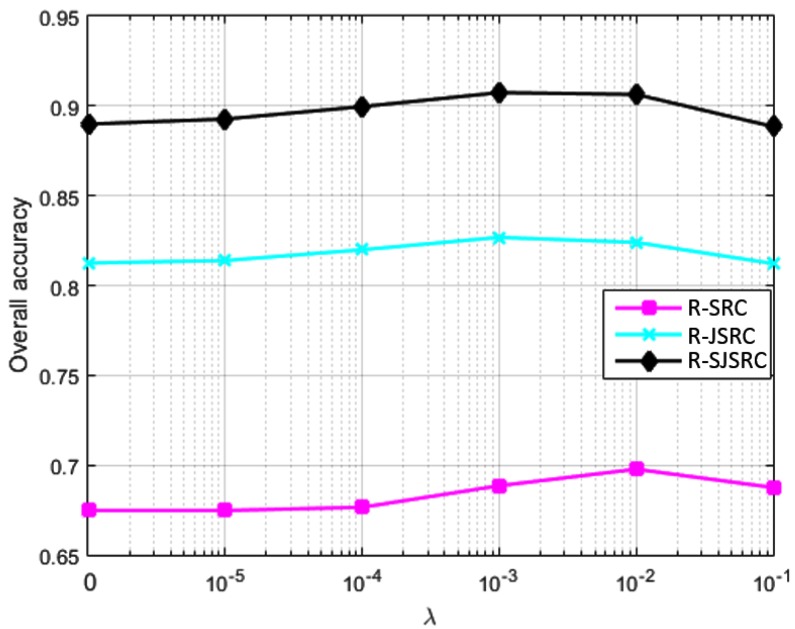
Classification performance of our methods with respect to parameter λ.

*Experiment 3 (Effect of sparsity constraint λ)*: In this experiment, we study the effects of the parameter λ on the classification performance for our methods. The test image was firstly degraded by Gaussian noise such that the SNR is 30dB, and then corrupted by sparse noise with $S_{b}=S_{p}=0.2$. The classification performance for R-SRC, R-JSRC and R-SJSRC is reported in Figure 4. Note that when the parameter λ is set as zero, R-SRC, R-JSRC and R-SJSRC reduce to SRC, JSRC and SJSRC, respectively.

We can observe in Figure 4 that the overall accuracies of the three models show similar trends in a function of the parameter λ. When the value of λ is relatively low, which means we enforce a smaller weight on the sparse noise, the performance of the proposed methods is not significantly improved over the results with λ=0. As the value of λ increases, the classification performance also improves, reaching its highest values at $\lambda =10^{-3}$ for R-JSRC and R-SJSRC, and at $\lambda =10^{-2}$ for R-SRC. The improvements for R-SRC, R-JSRC and R-SJSRC show the benefit of incorporating the effect of sparse noise in our models.

### 4.2. Results for Real HSI Experiment

In this section, two real HSI datasets are used: Indian Pines data set and an urban area HYDICE data set.

#### 4.2.1. Classification Results on the Real Datasets

**Figure 5:**
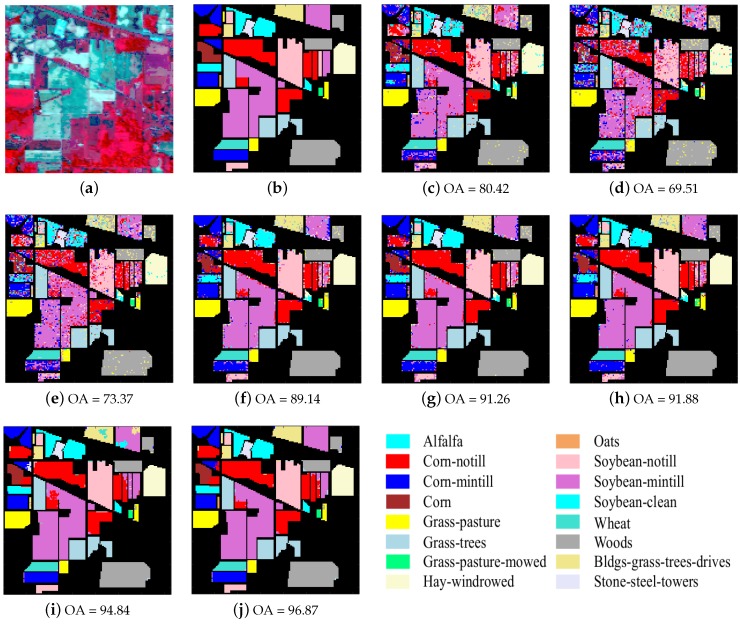
Indian Pines image. (**a**) false color image; (**b**) ground truth; and classification results (OA in percentage) obtained by (**c**) SVM; (**d**) SRC; (**e**) R-SRC; (**f**) JSRC; (**g**) R-JSRC; (**h**) NLW-JSRC; (**i**) SJSRC; (**j**) R-SJSRC.

The first experiment was conducted on the Indian Pines image, which was acquired by the Airborne/Visible Infrared Imaging Spectrometer (AVIRIS) sensor over the Indian Pines region in northwestern Indiana in 1992 as shown in Figure 5a. This image has 16 classes and 220 spectral reflectance bands ranging from 0.4 to 2.5 μm. In this experiment, 20 water absorption spectral bands in 104–108, 150–163 and 200 are removed; therefore, the real hyperspectral image size is 145×145×200. Nine percent of the labeled samples are randomly selected as training samples and the remainder as test samples as shown in Table 2.

The optimal parameters of our methods were: $\lambda =4\times 10^{-4}$, K=11 for R-SRC, $\lambda =1.5\times 10^{-3}$, $K_{0}=30,T=49$ for R-JSRC and $\lambda =0.003,K_{0}=50,Ns=300$ for R-SJSRC. For JSRC, the optimal window size was 7×7 and sparsity level was 30. In NLW-JSRC, the parameters were chosen from the recommendation of [21]. For SVM and SRC classifiers, we tuned the parameters such to produce the best classification results. The results are listed in Table 3 and Figure 5. In most cases, our method R-SJSRC yields better results than other classifiers. Based on super-pixel segmentation, the SJSRC model had at least 2.7% improvement over the reference methods JSRC and NLW-JSRC. Considering the sparse prior for multiple noise in the HSIs, our proposed R-SJSRC further improves OA by 1.5% over SJSRC. Moreover, the proposed robust models show a superior performance over SRC, JSRC and SJSRC, respectively. In Table 3, it should be noted that, even though the number of training samples for classes 1, 7 and 9 is very limited, both SJSRC and R-SJSRC still achieve a very high classification accuracy over others, which is largely due to the exploitation of super-pixel segmentation. Both R-SJSRC and SJSRC on a super-pixel level classification are able to alleviate the effect of unbalanced training samples on the performance to a certain degree.

We also test the computation time saving of R-SJSRC compared to R-JSRC. The experiment was implemented in Matlab R2015a on the computer with Intel Core i7-3930K CPU and 64 GB RAM, and recorded time consumption of one iteration including super-pixel segmentation and classification map generation for R-SJSRC and classification map generation for R-JSRC. The results show that R-JSRC spends 321 s, while R-SJSRC only takes 5 s for one iteration, which indicates the benefit of R-SJSRC in terms of time saving. The reason for the high complexity of R-JSRC mainly comes from the computation of sparse coefficient when using the sliding window, which has to be calculated multiple times.

**Figure 6:**
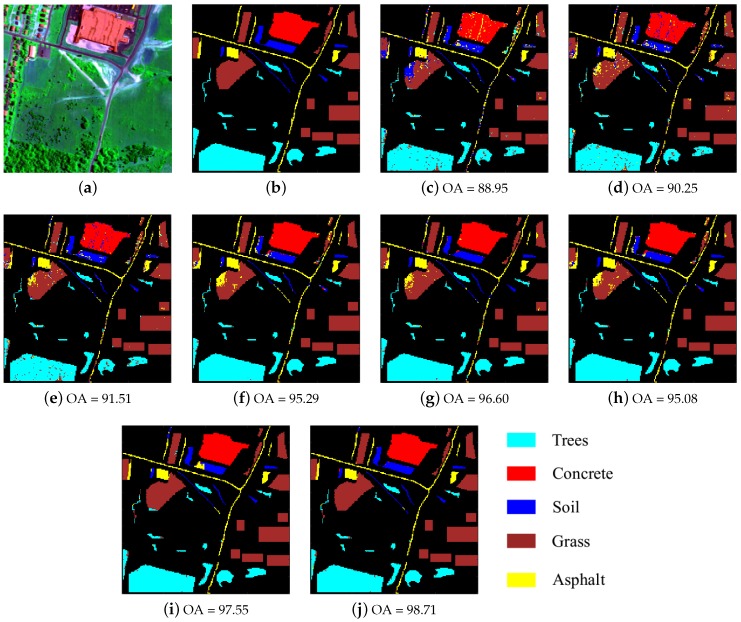
HYDICE Urban data. (**a**) false color image; (**b**) ground truth; and classification results (OA in percentage) obtained by (**c**) SVM; (**d**) SRC; (**e**) R-SRC; (**f**) JSRC; (**g**) R-JSRC; (**h**) NLW-JSRC; (**i**) SJSRC; (**j**) R-SJSRC.

The second image that we use for evaluation is HYDICE Urban captured by the HYDICE sensor [46]. The original image size is of 307×307×210 and there are five classes in total. In this experiment, we tested our method on a part of this image with size 200×200 as shown in Figure 6a. The number of bands was reduced to 188 by removing the bands 104–108, 139–151 and 207–210, which were seriously polluted by the atmosphere and water absorption. We used this image as it contains different types of noise including strips, dead lines, impulse noise and Gaussian noise [46]. The number of samples used for training and test are shown in Table 4.

The quantitative results and classification maps from different methods are shown in Table 4 and Figure 6. The optimal parameters of R-SRC, R-JSRC and R-SJSRC methods are obtained, respectively, by $\lambda =2.5\times 10^{-3},K=4$, $\lambda =0.01,K_{0}=10,T=25$ and $\lambda =0.01,K_{0}=12,Ns=1450$. For other classification methods, we tuned the parameters in order to yield the best results. The results in Table 4 and Figure 6 show clearly that the proposed R-SJSRC model performs better than other classification methods on the HYDICE Urban image in terms of quantitative measurements and visual evaluation. A superior performance can be also viewed for other robust models, i.e., R-SRC and R-JSRC, over SRC and JSRC.

#### 4.2.2. The Effect of the Training Sample Size

**Figure 7:**
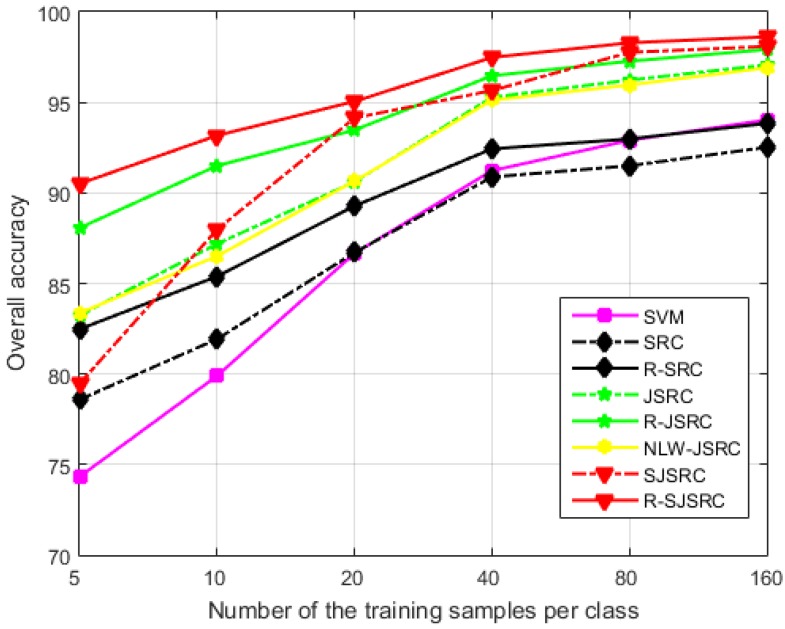
The influence of the training sample size on the performance of different methods. The test image is HYDICE Urban.

Here, we examine the effect of the training set size on the classification performance, using HYDICE Urban image as a case study. The number of training samples per class was set as 5, 10, 20, 40, 80 and 160, respectively, and the parameters for different methods were fixed as earlier specified. The results shown in Figure 7 reveal that the OA of all the methods gets improved significantly with the increase of training sample size, and R-SJSRC consistently achieves the best performance over all other tested methods. It can be observed that the highest improvement of R-SJSRC over SJSRC, as well as R-JSRC over JSRC and R-SRC over SRC is obtained when the number of training samples is the smallest (five per class). This improvement, resulting from accounting for the sparse noise in our model, turns out to be less significant when the size of the training set increases. This demonstrates that our robust model is especially effective when the training samples are limited.

#### 4.2.3. The Influence of the Segmentation Granularity

**Figure 8:**
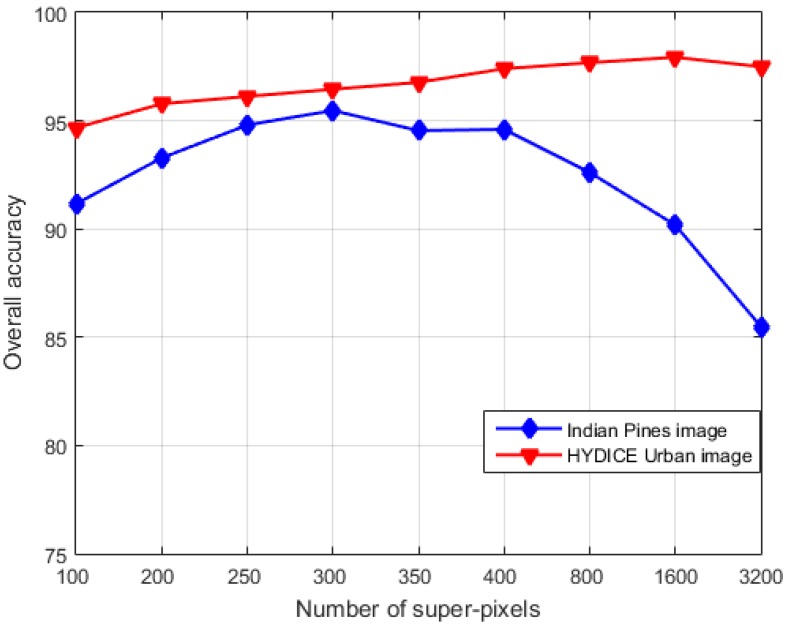
The effect of the number of super-pixels on the performance of R-SJSRC with two real HSIs: Indian Pines and HYDICE Urban.

To investigate the influence of the segmentation granularity on the performance of R-SJSRC, we conduct the experiments with varying number of super-pixels and record the resulting overall classification accuracy. Figure 8 shows the results for HYDICE Urban image and Indian Pines, where the number of super-pixel Ns is ranging from 100 to 3200, and the parameters of R-SJSRC are as specified earlier. The results demonstrate that the OA of HYDICE Urban image is less sensitive to the number of super-pixels than Indian Pines image for R-SJSRC. The OA of HYDICE Urban image stably increases to 97.93% when the value of Ns is less than 1600. The OA of Indian Pines image rapidly reaches to the top of 95.47% at Ns=300, and then drops down to 85% at Ns=3200. The reason for the stronger sensitivity of Indian Pines to Ns may be caused by the large diversity of the ground truth in the same class. When the number of super-pixels is large, more homogeneous regions will be separated into many small pieces, which results in the constraint relaxation of joint sparsity for the pixels in the same super-pixel and deteriorates the performance of R-SJSRC.

### 4.3. Practical Specification of the Parameters

In our experiments, we make sure that the comparison between different methods is fair by presenting for all of them the best achievable performance, assuming that the parameters were set optimally. In practice, ground truth data are rarely available. We advise the user in this case to optimize the parameters (using e.g., a widely adopted grid search) for images that are similar (in resolution and variability) to the ones being tested and for which ground truth data are available. The parameter values that we give may also be used without extensive decrease of the performance on a wide range of images of two types: AVIRIS and high-resolution urban images. The diagrams where we report the influence of the different parameters should also serve as a useful guideline in this respect. Figure 4 shows that λ can be chosen in a relatively wide range around the optimal value, without strongly affecting the performance. The same holds for the segmentation parameter, especially for Urban types of images.

## 5. Conclusions

In this work, we have proposed a robust classification model for HSIs, which combines an appropriate statistical model for the sparse noise and the representation coefficients of test samples into a unified framework, explicitly accounting for both Gaussian noise and sparse noise. An alternating minimization strategy is utilized to solve the resulting optimization problems. The robust model can easily generalize the off-the-shelf classification model to a robust version. The superior performance of the proposed methods over the existing methods is confirmed by the experiments on both real and simulated data, which demonstrates the effectiveness of the proposed robust model. 

## Figures and Tables

**Table 1:** Results for simulated data with different classifiers *.

Class	Class Name	Train	Test	SVM	SRC	R-SRC	JSRC	R-JSRC	NLW-JSRC	SJSRC	R-SJSRC
1	Roof	146	2770	0.7466	0.5842	0.5873	0.7727	0.7918	0.7790	0.7897	**0.7962**
2	Road	91	1728	**0.6742**	0.4100	0.4197	0.5219	0.5374	0.5204	0.5122	0.5425
3	Trail	64	1200	0.7585	0.6900	0.7070	0.7417	0.7540	0.7543	**0.9110**	0.9099
4	Grass	90	1700	0.8726	0.7536	0.7712	0.9463	0.9496	0.9468	0.9801	**0.9834**
5	Shadow	56	1064	0.7087	0.4234	0.4487	0.5778	0.5738	0.5617	0.8237	**0.8273**
6	Tree	65	1216	**0.7970**	0.4792	0.5042	0.5954	0.6038	0.5881	0.6846	0.7160
OA				0.7595 ±0.0095	0.5650 ±0.0087	0.5787 ±0.0101	0.7109 ±0.0142	0.7218 ±0.0148	0.7114 ±0.0156	$\underline{0.7792}$ $\underline{\pm 0.0208}$	0.7912 ±0.0192
AA				0.7596 ±0.0129	0.5567 ±0.0142	0.5730 ±0.0154	0.6941 ±0.0144	0.7017 ±0.0152	0.6917 ±0.0160	$\underline{0.7836}$ $\underline{\pm 0.0230}$	0.7959 ±0.0190
κ				0.7034 ±0.0119	0.4623 ±0.0123	0.4797 ±0.0140	0.6421 ±0.0174	0.6553 ±0.0182	0.6426 ±0.0192	$\underline{0.7284}$ $\underline{\pm 0.0258}$	0.7432 ±0.0232

* Abbreviations: support vector machines (SVM), sparse representation classification (SRC), robust SRC (R-SRC), joint SRC (JSRC), robust JSRC (R-JSRC), nonlocal weighted JSRC (NLW-JSRC), super-pixel level JSRC (SJSRC) and robust SJSRC (R-SJSRC). The best result in each row is denoted in bold and suboptimal result is underlined.

**Table 2:** Reference classes for the Indian Pines.

No.	Class Name	Train	Test
1	Alfalfa	6	40
2	Corn-notill	129	1299
3	Corn-mintill	83	747
4	Corn	24	213
5	Grass-pasture	48	435
6	Grass-trees	73	657
7	Grass-pasture-mowed	5	23
8	Hay-windrowed	48	430
9	Oats	4	16
10	Soybean-notill	97	875
11	Soybean-mintill	196	2259
12	Soybean-clean	59	534
13	Wheat	21	184
14	Woods	114	1151
15	Bldgs-grass-trees-drives	39	347
16	Stone-steel-towers	12	81
	Total	958	9291

**Table 3:** Overall classification accuracy for Indian Pines with different classifiers.

Class	SVM	SRC	R-SRC	JSRC [20]	R-JSRC	NLW-JSRC [21]	SJSRC	R-SJSRC
1	0.6275	0.4125	0.5075	0.5625	0.6350	0.5950	**0.9800**	**0.9800**
2	0.7807	0.6122	0.6546	0.8570	0.8780	0,8917	**0.9799**	0.9427
3	0.7106	0.5396	0.5750	0.8371	0.8541	0,8617	**0.9601**	0.9426
4	0.5362	0.3286	0.3770	0.6892	0.7469	0,7113	**0.9920**	0.8441
5	0.8968	0.8478	0.8678	0.9159	0.9292	**0,9366**	0.9172	0.9163
6	0.9534	0.9307	0.9470	0.9962	0.9970	0,9976	**1.0000**	0.9976
7	0.8130	0.7565	0.8261	0.6304	0.6652	0,6783	**0.9696**	**0.9696**
8	0.9584	0.9170	0.9598	0.9988	0.9993	**0,9995**	0.9977	0.9977
9	0.5813	0.5125	0.5813	0.4125	0.4750	0,6625	**1.0000**	0.8000
10	0.7506	0.6103	0.6466	0.8312	0.8519	0,8665	0.8574	**0.9271**
11	0.8053	0.7000	0.7291	0.8726	0.8977	0,9137	0.9099	**0.9508**
12	0.7315	0.5075	0.5772	0.8384	0.8936	0,9026	0.9296	**0.9700**
13	0.9544	0.9538	0.9668	**0.9967**	**0.9967**	**0,9967**	0.9951	0.9951
14	0.9308	0.9056	0.9145	0.9791	0.9815	**0,9856**	0.9569	0.9818
15	0.5545	0.4596	0.4937	0.7960	0.8499	0,8369	0.8939	**0.9677**
16	0.9346	0.8531	0.8605	0.9840	0.9852	**0,9938**	0.9790	0.9679
OA	0.8096 ±0.0066	0.7015 ±0.0039	0.7333 ±0.0024	0.8851 ±0.0047	0.9055 ±0.0040	0.9137 ±0.0064	$\underline{0.9407}$ $\underline{\pm 0.0008}$	0.9547 ±0.0095
AA	0.7825 ±0.0211	0.6780 ±0.0137	0.7178 ±0.0155	0.8248 ±0.0226	0.8523 ±0.0228	0.8644 ±0.0283	0.9574 ±0.0016	$\underline{0.9469}$ $\underline{\pm 0.0271}$
κ	0.7827 ±0.0074	0.6588 ±0.0043	0.6952 ±0.0026	0.8690 ±0.0053	0.8921 ±0.0046	0.9014 ±0.0074	$\underline{0.9325}$ $\underline{\pm 0.0009}$	0.9483 ±0.0109

**Table 4:** Overall classification accuracy for urban with different classifiers.

Class	Class Name	Train	Test	SVM	SRC	R-SRC	JSRC	R-JSRC	NLW-JSRC	SJSRC	R-SJSRC
1	Trees	30	3093	0.9251	0.9230	0.9269	0.9817	**0.9856**	0.9812	0.9691	0.9737
2	Concrete	30	1380	0.9696	0.9787	0.9874	0.9978	0.9990	0.9977	**1**	0.9999
3	Soil	30	607	0.8638	0.8359	0.8624	0.7685	0.8891	0.7802	0.8611	**0.9432**
4	Grass	30	4014	0.9055	0.8984	0.9208	0.9421	0.9590	0.9426	**0.9915**	0.9863
5	Asphalt	30	882	0.7832	0.7953	0.8194	0.9117	0.9179	0.9027	0.9621	**0.9711**
OA				0.9071 ±0.0123	0.9041 ±0.0139	0.9191 ±0.0114	0.9488 ±0.0086	0.9649 ±0.0049	0.9488 ±0.0083	$\underline{0.9752}$ $\underline{\pm 0.0003}$	0.9803 ±0.0058
AA				0.8894 ±0.0107	0.8863 ±0.0094	0.9034 ±0.0073	0.9204 ±0.0094	0.9501 ±0.0084	0.9209 ±0.0093	$\underline{0.9568}$ $\underline{\pm 0.0007}$	0.9748 ±0.0094
κ				0.8706 ±0.0166	0.8662 ±0.0189	0.8869 ±0.0156	0.9284 ±0.0119	0.9508 ±0.0069	0.9284 ±0.0115	$\underline{0.9651}$ $\underline{\pm 0.0004}$	0.9723 ±0.0081
